# Real-Time Indoor Environmental Quality (IEQ) Monitoring Using an IoT-Based Wireless Sensing Network

**DOI:** 10.3390/s24216850

**Published:** 2024-10-25

**Authors:** Tsz-Wun Tsang, Kwok-Wai Mui, Ling-Tim Wong, Angus Chun-Yu Chan, Ricky Chi-Wai Chan

**Affiliations:** 1 Department of Building Environment and Energy Engineering, The Hong Kong Polytechnic University, Hong Kong, China; 2 S.H.K. Real Estate Management Co., Ltd., Hong Kong, China

**Keywords:** indoor environmental quality, internet of things (IoT), human-centric management, real-time environmental monitoring

## Abstract

In recent years, our time spent indoors has risen to around 90% and to maintain an occupant’s comfort and well-being, Indoor Environmental Quality (IEQ) is monitored. Concerned with inhabitant’s satisfaction and health, the adoption of smart solutions for IEQ monitoring and improvement has expanded. The solution this study explores is an occupant-centric approach involving the implementation of an Internet of Things (IoT) IEQ sensing network in a prominent office skyscraper in Hong Kong. Over the course of 15 months, real-time IEQ data were collected from 12 locations within the building. The data were collected at 1-min time intervals and consisted of readings of indoor air temperature, radiant temperature, relative humidity, air velocity, carbon dioxide (CO_2_), particulate matter (PM_10_ and PM_2.5_), horizontal illuminance levels, and sound pressure levels, which served as the basis of the assessment made about the qualities of thermal comfort, indoor air quality (IAQ), aural comfort, and visual comfort. Compared to traditional periodic surveys, this IoT-based sensing network captured instantaneous environmental variations, providing valuable insights into the indoor environment’s spatial characterization and temporal dynamics. This smart solution also assisted facility management in terms of identifying sources of discomfort and developing effective mitigation strategies accordingly. This study presents an occupant-centric approach to improve occupant comfort and energy efficiency within office buildings. By customizing the built environment to enhance occupants’ well-being, comfort, and productivity, an emphasis is placed on a more personalized and occupant-focused design strategy. This approach integrates technical design with human experience, highlighting the importance of real-time physical and subjective surveys for achieving optimal results.

## 1. Introduction

During the construction and operational lifespan of a building, importance is often placed on ensuring optimal Indoor Environmental Quality (IEQ) standards to safeguard the health and well-being of its inhabitants. Guided by international benchmarks such as ASHRAE 189.1 [1], the assessment of a building’s IEQ includes critical aspects such as acoustics, indoor air quality (IAQ), and thermal comfort. These factors are pivotal in maintaining an indoor environment that not only promotes health but also enhances productivity [2].

The impact of poor IEQ varies with location and occupants’ behavior, yet a prevailing consensus suggests that buildings falling short in IEQ often subject individuals to discomfort, ranging from mild symptoms like headaches and nausea to more severe conditions such as sick building syndrome (SBS) [3,4]. Beyond direct health concerns, poor thermal comfort, elevated noise levels, and inadequate exposure to light contribute significantly to distractions, poor work performance, and compromise overall comfort. Research has indicated that noise pollution alone can lead to a substantial 66% decline in productivity among workers [5], while higher temperatures have been linked to heightened stress levels among occupants [6].

While meeting stringent IEQ standards may seem to pose challenges in terms of increased costs and energy consumption, studies [7] have demonstrated that adherence to these standards can be achieved through the implementation of energy-efficient technologies and systematic testing protocols. Moreover, the long-term benefits in terms of enhanced productivity and improved health outcomes often justify the initial financial considerations.

Recognizing the multifaceted impact of IEQ on occupants and operational efficiency, a human-centric approach emerges as a compelling strategy to elevate IEQ standards. By prioritizing the perspectives and needs of building occupants in design decisions, a built environment can be tailored to optimize their health, comfort, and productivity. Instances where occupants’ feedback has been disregarded underscore the critical importance of aligning technical design with human experience [8], emphasizing the indispensable role of comprehensive field surveys in realizing optimal outcomes.

Through the implementation of field surveys, sensors have the capacity to gather quantitative data on specific parameters that represent various comfort categories. These data are assessed in parallel with user feedback to determine the necessary improvements or changes needed to create the desired indoor environment. Presently, the conventional approach to IEQ monitoring through periodical assessment necessitates extensive manual effort and engagement during data collection. Moreover, it requires professionals for device calibration, setup, maintenance, and dismantling [9]. Notably, post-survey disassembly mandates the manual transfer of each sensor’s locally stored data to an external system for subsequent data evaluation and analysis.

To overcome the limitations associated with traditional IEQ monitoring methods, many have opted to leverage Internet of Things (IoT) IEQ wireless sensing networks. An IoT-based sensing network can capture real-time environmental fluctuations influenced by outdoor weather patterns or human behavior. While IoT sensors may have lower precision than traditional laboratory-grade instruments, their value lies in providing a holistic perspective of environmental conditions due to higher sampling point density at a much lower implementation cost. This contemporary technology seamlessly transfers recorded measurements to a virtual cloud [10,11], enabling universal data accessibility via an internet connection. Despite occasional technical glitches such as signal intervention or data lost, continuous field monitoring facilitated by IoT yields more precise outcomes compared to the traditional technologies used currently [12].

In this vein, this study deployed an IoT IEQ monitoring system in one of Hong Kong’s prominent skyscrapers in an effort to enhance the well-being of its occupants amidst urban stress and tropical climate challenges. Once implemented, this system collected and transmitted real-time environmental data wirelessly to a backend cloud server. Emphasizing a human-centric approach, this server linked with an interactive web platform where occupants could access and contribute feedback on their perceived thermal comfort and IAQ. This study presents an implementation framework for long-term real-time IEQ monitoring, aiming not only to highlight the significance of IEQ and improve the transparency of environmental conditions, but also to enhance occupants’ engagement in IEQ control and maintenance [13].

## 2. Materials and Methods

### 2.1. Study Location and Measurement Campaign

In this study, a state-of-the-art IoT IEQ monitoring system was installed in a renowned Hong Kong skyscraper constructed over four decades ago. Over the years, the building has embraced sustainable practices through various initiatives, including enhancements to critical systems like chillers and air handling units (AHU), the adoption of energy-efficient LED lighting, and the implementation of a Building Management System (BMS) to promote energy efficiency. Recognizing a plateau in hardware upgrades, management pivoted towards “soft” management by addressing the human-centric aspect through the introduction of a cutting-edge IoT IEQ monitoring system.

The measurements were collected from 12 locations over a 15-month period, starting in January 2023 until March 2024. The systems were distributed across four floors, including three regular commercial office floors and one basement floor serving as the engineering department’s workshop. During the initial set-up period, each wireless system was strategically placed at the center of their respective zones served by an individual AHU. This placement was intentional, considering that each AHU operates independently, catering to a specific group of building occupants within the zone. In addition, these systems were positioned near staff working areas within the sitting breathing zone to more accurately record the environmental conditions influenced by human activities.

### 2.2. IEQ Monitoring System

A technical consulting firm locally manufactured the established IoT-based sensing network (EVQSense, Hong Kong, China) to meet specific sensor requirements. The electrical board and sensors were sourced from the market, and data calibration was performed by comparing raw sensor data with laboratory-grade IEQ instruments. The system was deployed in a fixed location with a steady power supply and operated at a sampling frequency of 1 min. Additionally, an internal battery was integrated to support measurements at 1 min intervals for up to 4 h in case of power shortages. This initiative was a part of the zone renovation efforts to enhance overall IEQ. It captured instantaneous environmental variation induced by day-to-day human activities or outdoor weather. The readings collected also provided spatial characterization and temporal understandings of an environment, which aided in identifying sources of discomfort and mitigation strategies. The indoor parameters that were assessed to draw these conclusions included the following:

- Air temperature;
- Radiant temperature;
- Relative humidity;
- Air velocity;
- Carbon dioxide (CO_2_);
- Particulate matter (PM_10_ and PM_2.5_);
- Horizontal illuminance level;
- Sound pressure level.

**Figure 1:**
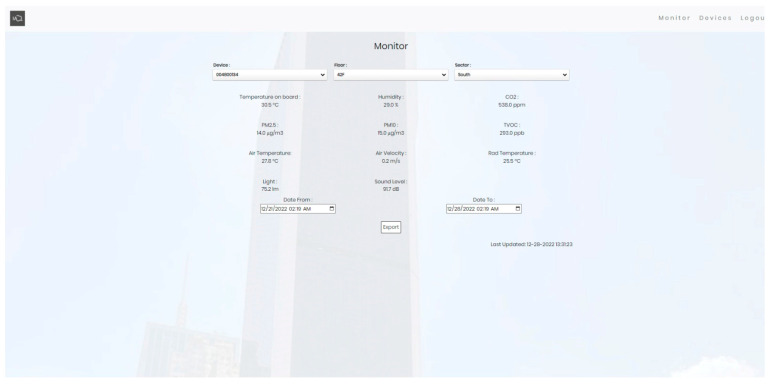
Screenshot of the user web-based portal offering real-time statistics about the environment.

During the measurement period, all data were transmitted wirelessly through a Wi-Fi network and stored in a backend cloud server. As shown in Figure 1, these live results were accessible to the building occupants through a designated user-interactive web-based/mobile application platform, where they could review the data and record their subjective comfort sensations and acceptance regarding the thermal environment and IAQ. Each individual sensor’s specification of the IEQ sensing network is exhibited in Table 1.

## 3. Results and Discussion

### 3.1. Air Temperature and Radiant Temperature

Below, Table 2 outlines the mean range of air temperature and the mean radiant temperature measured at 12 sampling zones within the building during workdays from 9 a.m. to 5 p.m. However, it is important to note that data collected on 17 July 2023 and 1 September 2023 were excluded from the analysis due to work suspension caused by typhoons. Notably, typhoons with heavy rainfall can impact indoor environmental conditions, leading to elevated relative humidity levels. Nevertheless, these effects were not evident in the data gathered over those two days.

The indoor air temperature and mean radiant temperature remained stable across different zones throughout the measurement period. The mean radiant temperature exhibited larger daily fluctuations than the air temperature, possibly due to factors such as solar radiation. Spatial variation was observed, with the 42F-North zone having the lowest mean air temperature (22.8 °C) and the 10F-South-East zone having the highest (25.6 °C). Similarly, the mean radiant temperature was lowest at 42F-North (21.7 °C) and highest at 10F-South-East (24.7 °C). On average, across all 12 zones, the mean air and radiant temperatures were 24.4 °C and 23.5 °C, respectively.

Furthermore, when examining the data collected on weekends and holidays, a linear regression analysis was conducted to establish the outdoor–indoor temperature relationship for each zone. Each relationship follows Equation (1), where T_a_ is the indoor air temperature and T_o_ is the outdoor temperature, but varies between respective co-efficient C_1_ and constants C_0_, which can be found in Table 3.

(1) $$T_{a}=C_{1}\times T_{o}+C_{0}$$

These relationships allowed comparisons to be made between the indoor climate, which was regulated by the Mechanical Ventilation and Air Conditioning (MVAC) system and the outdoor temperature—comparisons that could quantify estimated cooling loads. Within these calculations, because the B2-Workshop operates all the time, the indoor air temperature without MVAC control could not be estimated.

#### 3.1.1. Adaptive Comfort Temperature (ACT) Control Models

To derive a relationship between indoor design temperatures and exterior meteorological/climatological parameters, the adaptive comfort temperature (ACT) control model was used. Specific to Hong Kong, Equation (2) remains the model used under the findings measured in 55 office buildings, where T_n_ is the adaptive comfort temperature and T_o_ is the outdoor temperature [14]:

(2) $$T_{n}=0.158\times T_{o}+18.303$$

**Figure 2:**
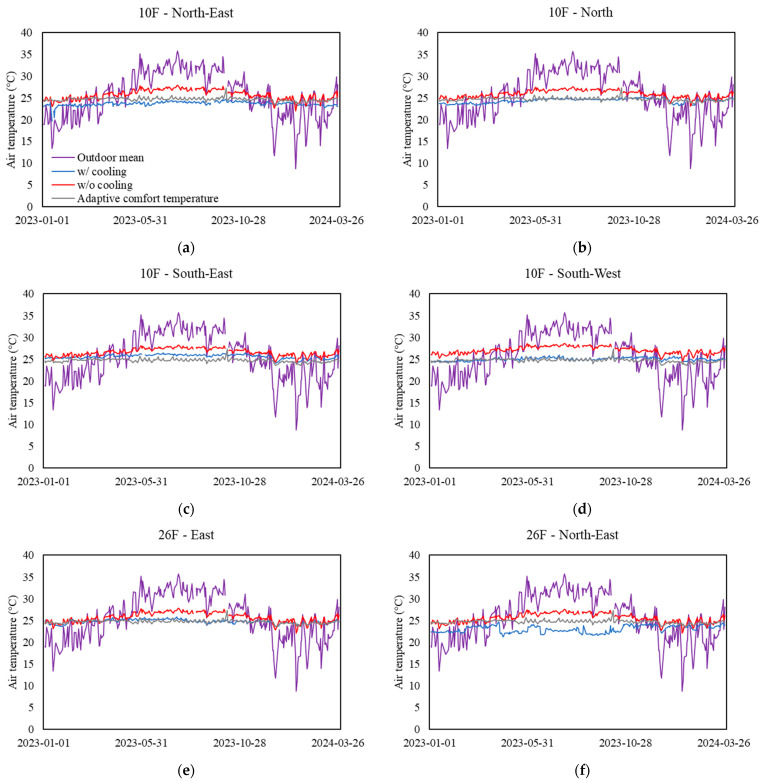

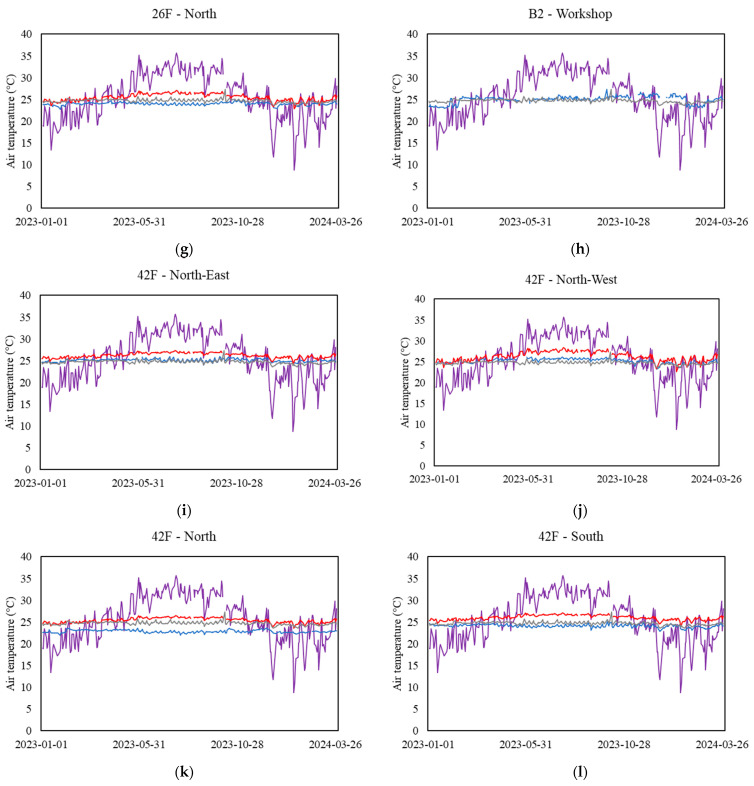
Daily mean outdoor temperature and indoor air temperature at sampling zones. (**a**–**d**) Measurements recorded from the 10th floor, (**e**–**g**) measurements recorded from the 26th floor, (**h**) measurements recorded from the basement floor, and (**i**–**l**) measurements recorded from the 42nd floor.

Figure 2 illustrates the daily mean outdoor temperature, the predicted ACT based on outdoor temperature, the indoor air temperature without cooling predicted by the linear regression equations, and the measured indoor air temperature with MVAC control at various sampling zones. The data cover the 15-month measurement period from 9 a.m. to 5 p.m. on workdays.

During the periods from January to early April 2023 and from mid-December 2023 to the end of March 2024, the cooling requirement was generally less intense when the outdoor temperature was relatively low. The highest cooling demand was observed between mid-May and the end of October 2023, as the outdoor temperature reached 30–35 °C, resulting in an indoor air temperature without MVAC control peaking at 28.6 °C.

#### 3.1.2. Average Daily Cooling Requirements

**Figure 3:**
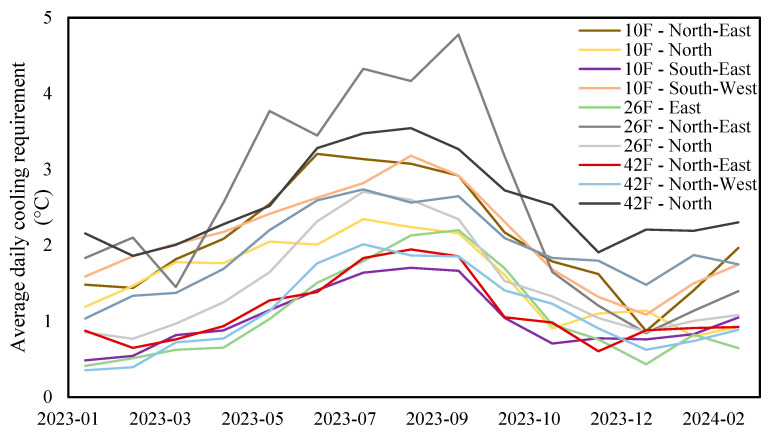
Average daily cooling requirement of each zone.

Below, Figure 3 presents the average daily cooling requirement of each zone over the 15 months. Of all the zones, 42F-North exhibited the highest average daily cooling demand, with an annual average of 2.55 °C. However, during the summer season, 26F-North-East stood out with the highest cooling requirement. In September 2023, it peaked at 4.8 °C, indicating the need for intensive cooling during hot weather conditions.

In contrast, zones such as 42F-North-East, 42F-North-West, 26F-East, and 10F-South-East demonstrated consistently lower cooling demands. These zones had an annual peak of less than 2.5 °C and a yearly average below 1.2 °C, on account of these areas potentially experiencing reduced heat gain or being furnished with superior thermal insulation.

### 3.2. Energy Saving Potential by Set-Point Temperature Adjustment

#### 3.2.1. Adaptive Comfort Temperature

As shown in Figure 2f,k, both the 26F-North-East zone and 42F-North zone recorded indoor air temperatures as being lower than the ACT for 32% of the time across the testing period. On these days, the indoor air temperatures were, on average, 0.85 °C and 0.83 °C lower than the ACT, respectively, indicating that these zones were overcooled. Energy savings could be achieved by adjusting the set-point temperature of these zones to be above the ACT.

Integral to this discussion is the recognition that although the indoor air temperatures in other zones consistently remained above the ACT, no complaints regarding the thermal environment were received throughout the entire measurement period. This result suggests that maintaining an indoor air temperature higher than the ACT may be possible without compromising occupants’ thermal comfort.

#### 3.2.2. Mean and Maximum Indoor Air Temperature of Each Zone

Alternatively, this second strategy stems from the absence of reported thermal discomfort during the measurement period and the assumption that the occupants of the building have similar thermal comfort sensations and expectations. We propose that the mean air temperature of each zone can serve as a reference for setting the minimum set-point temperature, while the maximum temperature recorded can be considered the threshold for thermal discomfort. Table 4 presents the energy-saving potential if the set-point temperature is adjusted to the mean and maximum air temperature of each zone accordingly.

By implementing these set-point temperature adjustment strategies based on the average daily cooling requirements and the thermal comfort levels of each zone, energy savings could be achieved while maintaining a comfortable indoor environment for the occupants.

### 3.3. Carbon Dioxide (CO_2_) Levels and Energy Saving Potentials by Fresh Air Supply Adjustment

**Figure 4:**
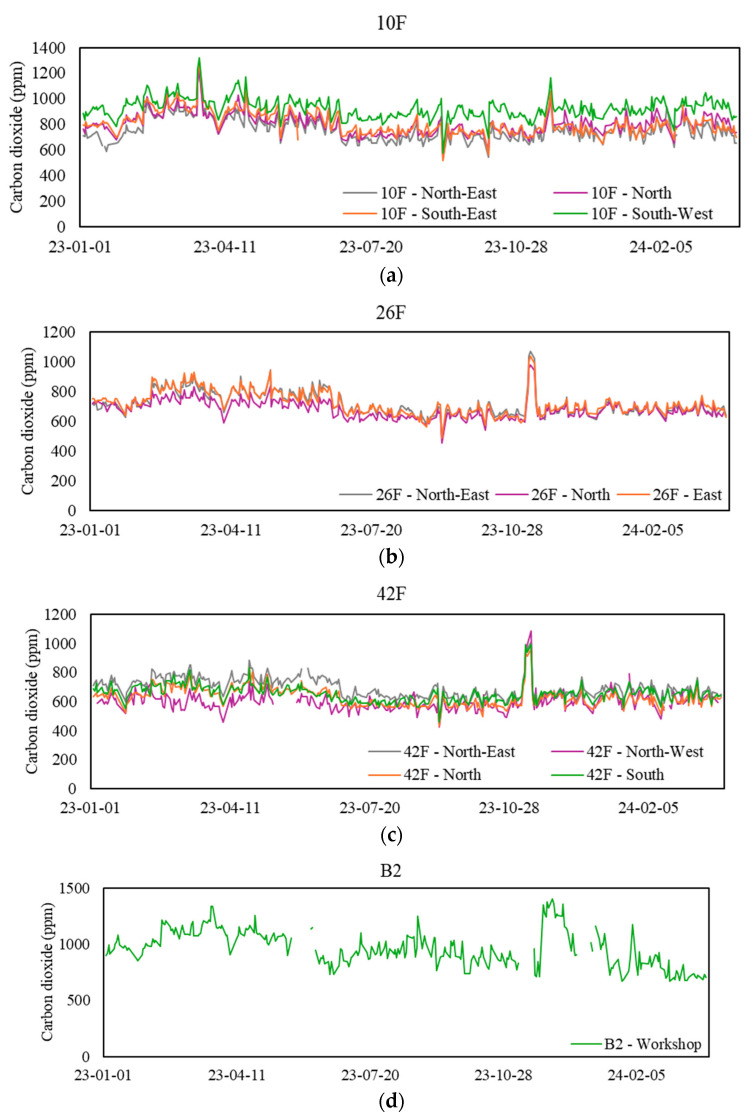
Daily mean carbon dioxide (CO_2_) levels at sampling zones. (**a**) Values for the 10th floor, (**b**) values for the 26th floor, (**c**) values for the 42nd floor, and (**d**) values for the basement floor.

Table 5 presents the mean range and statistics of CO_2_ levels at the 12 sampling zones during workdays from 9 a.m. to 5 p.m. The daily profile of CO_2_ generally exhibits two distinctive peaks around 1:30 p.m. and 6 p.m. In consideration of the 8 h average of CO_2_ levels, most zones had CO_2_ levels below 1000 ppm, which is the Good Class standard according to the IAQ Certification Scheme. However, this was not the case for 10F-South-West and B2-Workshop, as they both exceeded the exposure limit 15% and 40% of the time, respectively. Conversely, some zones consistently maintained CO_2_ levels below 800 ppm, including 26F-North and all zones belonging to 42F. Energy savings could be achieved in these zones by reducing the fresh air supply. Figure 4 plots the daily mean CO_2_ levels at various sampling zones during workdays from 9 a.m. to 5 p.m.

### 3.4. Relative Humidity

**Figure 5:**
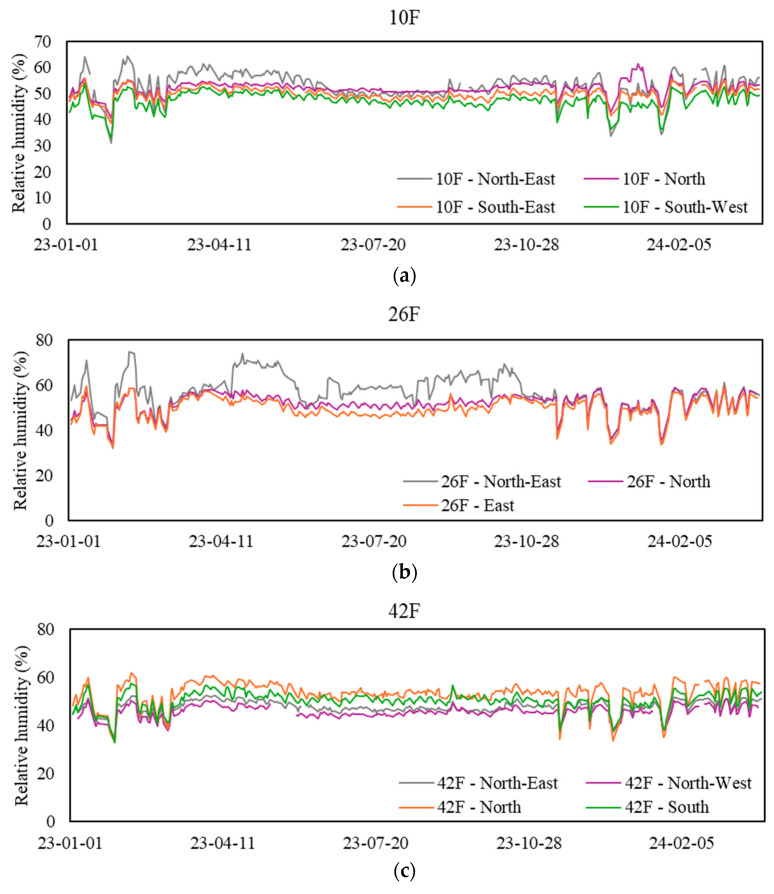

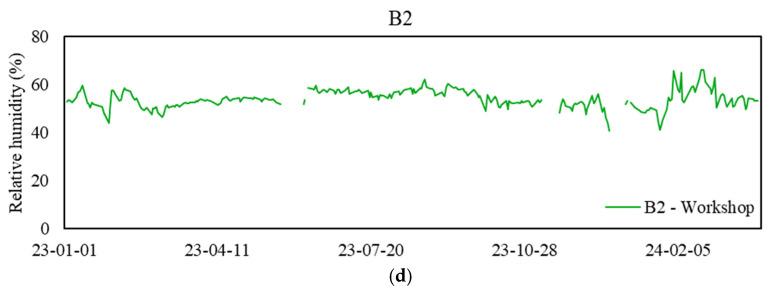
Daily mean relative humidity levels at sampling zones. (**a**) Values for the 10th floor, (**b**) values for the 26th floor, (**c**) values for the 42nd floor, and (**d**) values for the basement floor.

Table 6 presents the mean range and statistics of relative humidity at the 12 sampling zones during workdays from 9 a.m. to 5 p.m. When considering the 8 h average levels, most zones maintained a level below 60%, indicating a favorable humidity range. However, 26F-North-East remains the one exception, exhibiting poor humidity control during the summer, as evidenced by this sensor’s humidity levels exceeding the limit of 30% of the time. Figure 5 illustrates the daily mean relative humidity levels at various sampling zones during workdays from 9 a.m. to 5 p.m.

### 3.5. Particulate Matter (PM_10_ and PM_2.5_)

**Figure 6:**
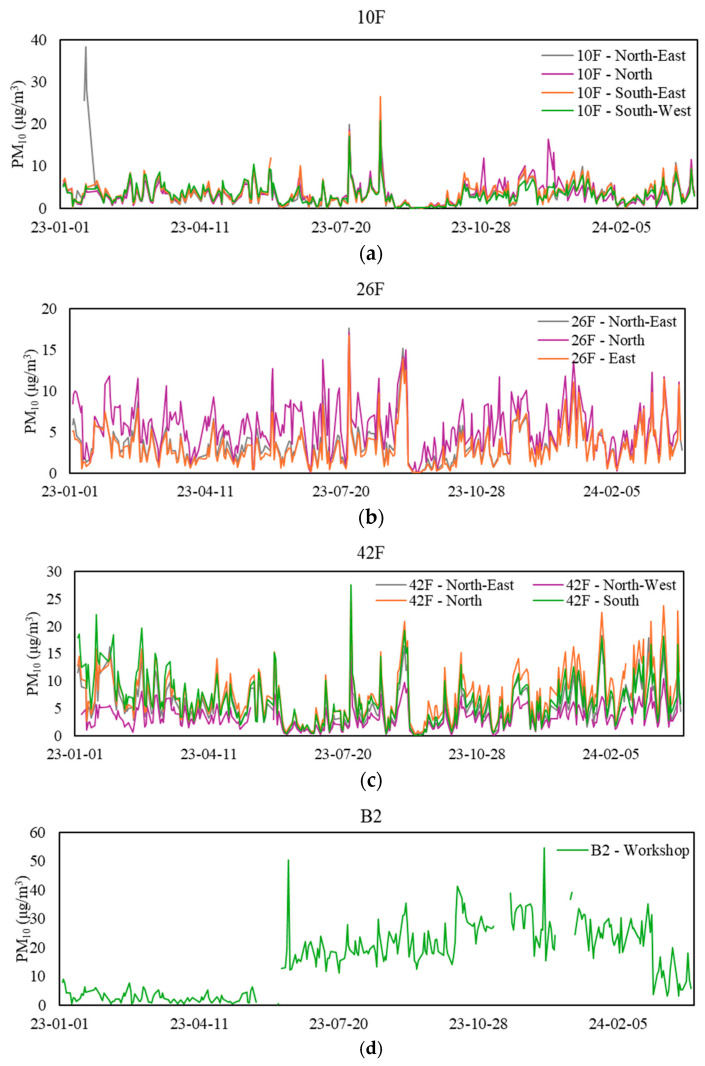
Daily mean PM_10_ levels at sampling zones. (**a**) Values for the 10th floor, (**b**) values for the 26th floor, (**c**) values for the 42nd floor, and (**d**) values for the basement floor.

**Figure 7:**
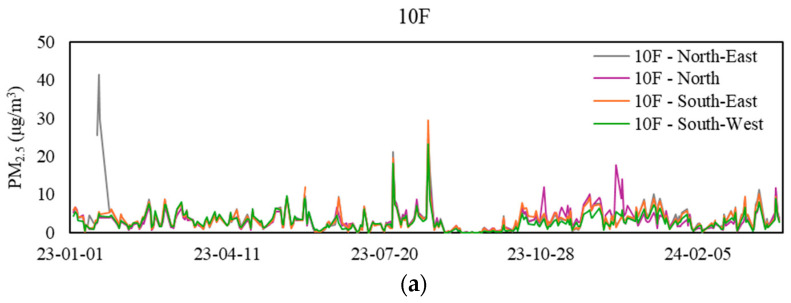

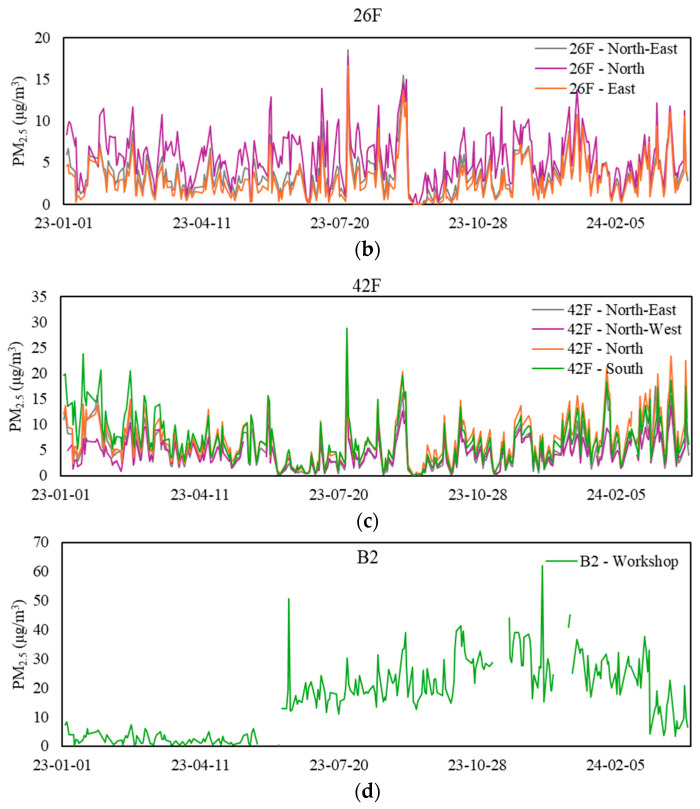
Daily mean PM_2.5_ levels at sampling zones. (**a**) Values for the 10th floor, (**b**) values for the 26th floor, (**c**) values for the 42nd floor, and (**d**) values for the basement floor.

Table 7 displays the mean range and statistics of PM_10_ and PM_2.5_ levels at the 12 sampling zones during workdays from 9 a.m. to 5 p.m. The daily profile of PM generally exhibits two distinctive peaks: the first around 9 am when the MVAC system starts, and the second during lunch at approximately 2:30 p.m. When considering the 8-h average, most zones maintained levels below 20 μg/m^3^, meeting the Excellent Class standard of the IAQ Certification Scheme (applicable to PM_10_ only). Contrary to this, one of the zones, B2-Workshop, exceeded the limit 37% of the time. None of the zones surpassed the Good Class exposure limit of 100 μg/m^3^ (applicable to PM_10_ only), indicating a good working environment with minimal levels of respirable suspended dust. Figure 6 and Figure 7 showcase the daily mean PM_10_ and PM_2.5_ levels at various sampling zones during workdays from 9 a.m. to 5 p.m.

### 3.6. Sound Pressure Levels (SPL)

**Figure 8:**
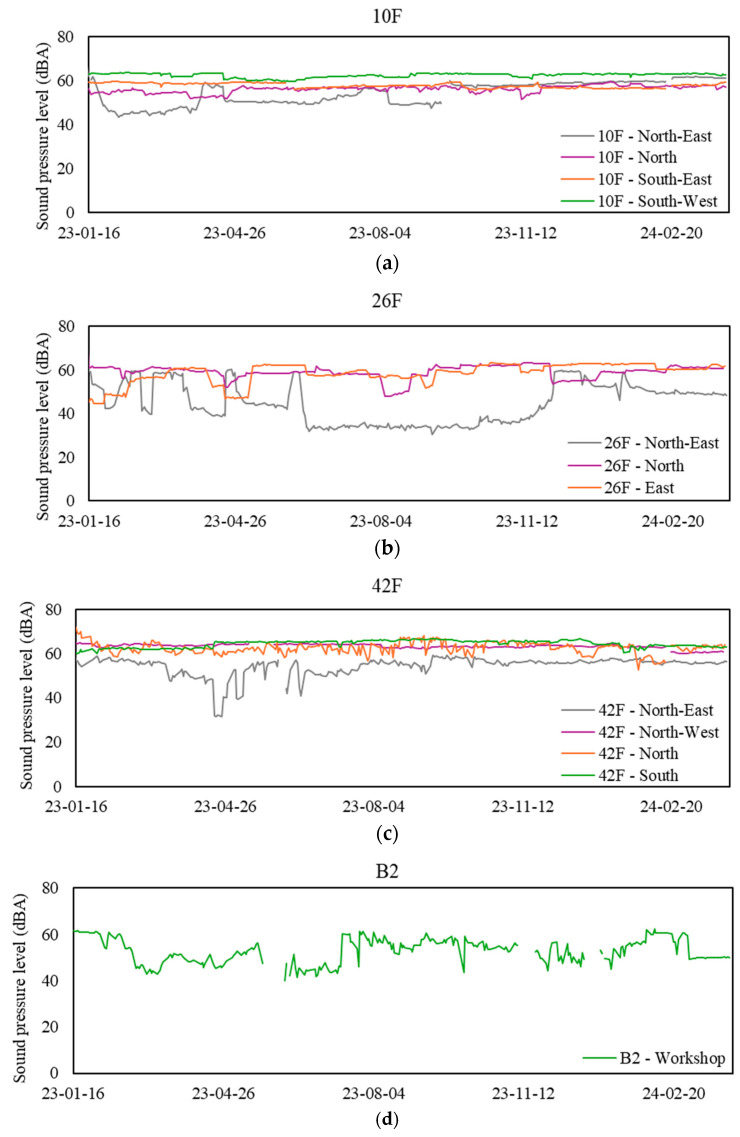
Daily mean sound pressure levels (SPL) at sampling zones. (**a**) Values for the 10th floor, (**b**) values for the 26th floor, (**c**) values for the 42nd floor, and (**d**) values for the basement floor.

Table 8 presents the mean range and statistics for SPLs at the 12 sampling zones during workdays from 9 a.m. to 5 p.m. Notably, the SPL measurements collected over the past 15 months indicate a consistently high level of sound within the office environment. This remains an exception to the data recorded from the first 10 workdays excluded from analysis due to their non-viability. Most sampling zones exhibited a mean daily SPL above 55 dBA, which serves as the threshold for an office environment. Following this, further investigation is necessary to identify the source of the elevated SPLs. On the contrary, such an abnormal observation of SPLs in an office setting may indicate technical issues with the SPL sensor, necessitating inspection, recalibration, or repair of the sensor. Figure 8 illustrates the daily mean SPLs at various sampling zones during workdays from 9 a.m. to 5 p.m.

### 3.7. Horizontal Illuminance Levels

**Figure 9:**
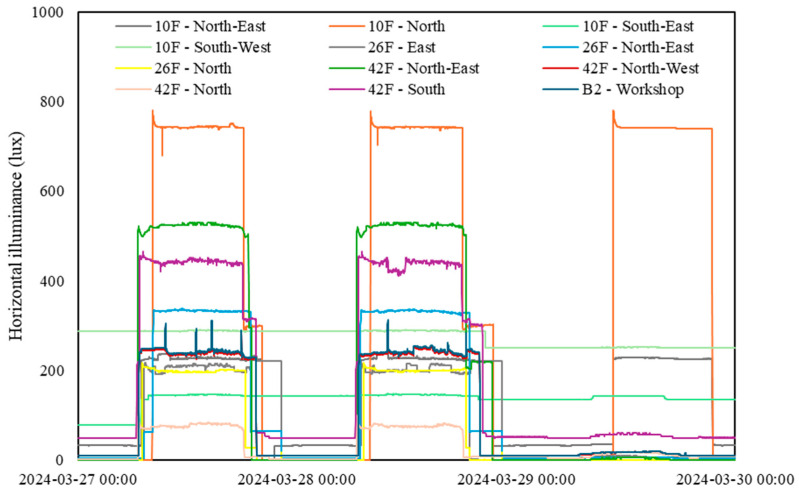
Typical daily profile of horizontal illuminance levels (light).

**Figure 10:**
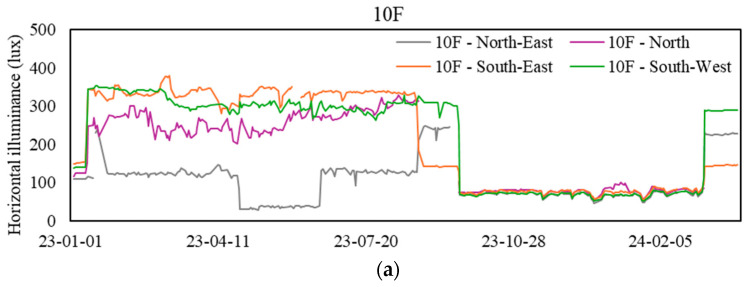

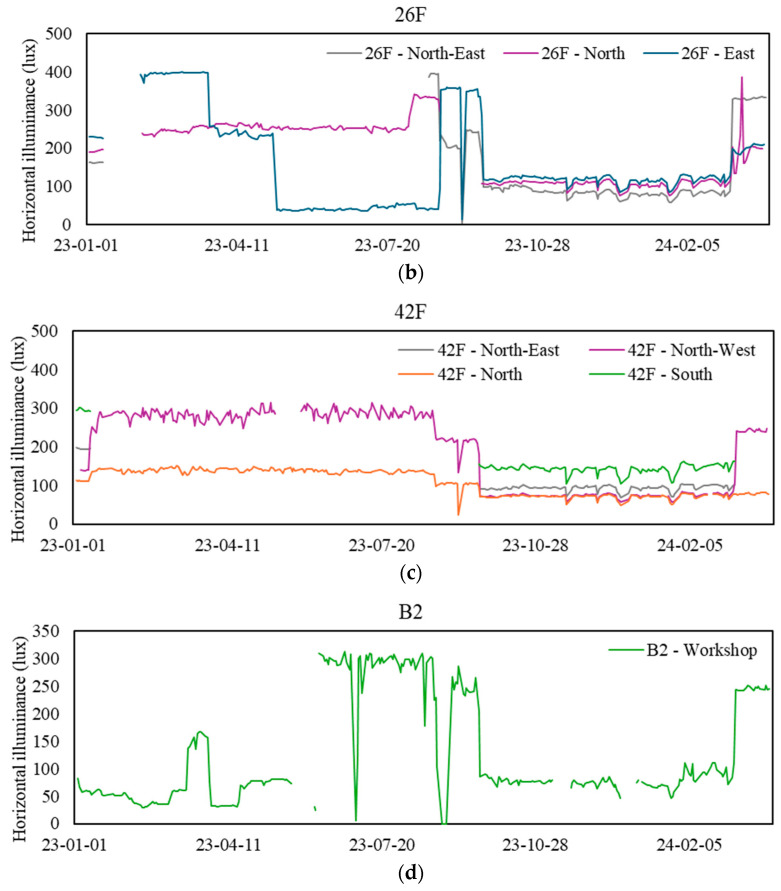
Daily mean horizontal illuminance (light) levels at sampling zones. (**a**) Values for the 10th floor, (**b**) values for the 26th floor, (**c**) values for the 42nd floor, and (**d**) values for the basement floor.

Figure 9 presents a typical daily profile of light levels after modifying the light-sensing device in March 2024. Due to the modification and subsequent transition period (September 2023 to March 2024), the data collected during this time were not suitable for analysis. Therefore, this figure provides a reference for recent light-level trends. Figure 10 displays the daily mean light levels at various sampling zones during workdays from 9 a.m. to 5 p.m. Figure 9 illustrates that only a few zones managed to sustain horizontal illuminance levels exceeding 500 lux, a criterion commonly adopted in current design practices. A local study focusing on acceptable illumination levels for office occupants revealed that a horizontal illuminance level of 518 lux is essential to uphold an 86% satisfaction rate regarding visual comfort [15]. It is necessary to consider that adaptive behaviors, like using desk lamps, may lead to underestimating the insufficiency of light levels for desk work, especially if no complaints have been registered. However, it is recommended that facility management look deeper into the lighting provision with regard to the particular task at hand in each zone.

### 3.8. Limitations and Future Development

Several limitations were identified in this study. Firstly, VOCs were initially among the targeted sampling parameters, and a TVOC IoT sensor was integrated into the system. However, during testing, it became evident that the sensors could not provide data that aligned well with laboratory-grade instruments. Given that VOCs are suspected to be significant contributors to sick building syndrome (SBS) [16,17], future IoT-based IEQ assessments should include monitoring VOCs using improved IoT sensors. Alternatively, statistical methods such as the likelihood ratio could be employed to forecast the risk of elevated TVOC levels based on surrogate IAQ parameters like PM and CO_2_, along with IAQ data gathered using traditional methods [18,19].

Secondly, despite the attempt to collect subjective sensation and satisfaction responses from the building occupants using an interactive platform, no feedback or complaints were obtained throughout the sampling period. For future implementations, to gather more subjective data for analysis and occupant-centric control, it is recommended that occupants are prompted regularly with IEQ questions on their computer screens or smart phones [20].

It is crucial to highlight that occupants often employ behavioral adjustments to enhance their thermal comfort when the thermal environment surpasses acceptable limits. These adaptive actions may include modifying clothing, using a paper fan, adjusting posture or hairstyle, and consuming hot or cold beverages [21,22,23]. Such behaviors can alleviate thermal discomfort resulting from the perceived environment—actions that cannot be captured by environmental measurements or reflected in subjective sensations or satisfaction assessments, but that impact energy consumption. While this study relied on environmental data and the condition of “no complaints imply an acceptable condition” to explore practical energy efficiency policies, there is merit in observing occupant adaptive behavior to refine these strategies further.

## 4. Conclusions

The study conducted at a prominent office skyscraper in Hong Kong using a state-of-the-art IoT-based sensing network provided valuable insights into the building’s performance, occupant comfort, and the MVAC system’s energy-saving potential.

In contrast to traditional environmental monitoring methods that rely on periodic surveys, this IoT-based sensing network can capture instantaneous environmental variations triggered by outdoor weather conditions or human activities. While IoT sensors may not provide measurements as precise as traditional laboratory-grade instruments, their strength lies in offering an overall view of environmental conditions. This system gives spatial characterization and temporal insights into the environment and assists in pinpointing sources of discomfort for devising effective mitigation strategies. Consequently, employing an IoT-based IEQ assessment enables real-time continuous monitoring with a significant reduction in implementation costs.

The key findings and recommendations are summarized below:

*Thermal comfort*

- The indoor air temperature and mean radiant temperature remained stable across different zones.
- Two zones, 26F-North-East and 42F-North, were found to be overcooled, suggesting that potential energy savings could be made by adjusting their set-point temperatures.
- Other zones maintained indoor air temperatures above the acceptable comfort range without any reported discomfort, indicating the possibility of raising the set-point temperatures for energy conservation.
- Most zones maintained desired humidity levels below 60%, except for the 26F-North-East zone during the summer season when humidity exceeded the limit for 30% of the time.

*Indoor air quality (IAQ)*

- Most zones maintained CO_2_ levels well meeting the Good Class standard, except for 10F-South-West and B2-Workshop, which had a substantial amount of time exceeding the exposure limit.
- Fresh air supply can be reduced in zones with consistently low CO_2_ levels (e.g., the 26F-North and 42F zones) to achieve energy savings.
- PM levels remained within the acceptable range, except in B2-Workshop, where PM levels exceeded the limit for a sizeable portion of the time.

*Aural environment*

- The sound pressure measurements consistently indicated high noise levels in the office environment, requiring further investigation into the source of elevated noise.
- Technical issues may arise concerning the quality of the SPL sensor, and re-calibration or repairs may be required.

*Visual environment*

- A detailed assessment of lighting provisions tailored to the specific tasks within each zone shall be conducted to ensure optimal lighting conditions that meet the visual comfort needs of office occupants and enhance overall productivity and well-being in the workspace.

The study highlights the importance of occupant-centric engineering approaches, real-time monitoring, and data-driven analysis to optimize building performance, enhance occupant comfort, and achieve sustainability goals. More specifically, by the implementation of this IoT IEQ monitoring system and maintaining a human-centric approach toward building environment changes, the process has achieved clearer communication and data transparency between the current inhabitants and the building estate. Through a web-based/mobile application platform accessible to all, occupants can conveniently report their subjective comfort sensations and satisfaction levels concerning the thermal environment and IAQ. When combined with physical data, this subjective comfort data can inform MVAC control decisions in response to occupant discomfort. By leveraging this human-centric approach, the system can dynamically adjust the indoor temperature set point to cater to the occupants’ preferences effectively. This combined effect generated targeted techniques to improve IEQ while reducing energy consumption.

## Figures and Tables

**Table 1:** Specification of the Indoor Environmental Quality (IEQ) sensing network.

Parameter	Measurement Range	Accuracy	Resolution
Air temperature	0 to 100 °C	±≤1 °C	0.1 °C
Radiant temperature	5 to 50 °C	±2 °C	0.1 °C
Relative humidity	0 to 100%	±≤2%	1%
Air velocity	0.2 to 20 m/s	±5% of reading	0.1 m/s
Carbon dioxide (CO_2_)	0 to 5000 ppm	±50 ppm	5 ppm
Particulate matter (PM_10_ and PM_2.5_)	0 to 999 μg/m^3^	±15 μg/m^3^	1 μg/m^3^
Horizontal illuminance level	0 to 40,000 lux	±5%	1 lux
Sound pressure level	35 to 130 dB	±3 dB	0.1 dB
System module			
Size dimensions	400 × 200 × 100 mm^3^		
Data transmission	2.4 GHz transmission with WSP2 security		

**Table 2:** Mean range and statistics of air temperature and radiant temperature at sampling zones.

	Air Temperature		Radiant Temperature	
Location	Mean Range (°C)	Mean (std) (°C)	Mean Range (°C)	Mean (std) (°C)
10F-North-East	19.7–24.8	23.7 (0.5)	19.1–24.2	22.5 (0.7)
10F-North	23.3–25.2	24.4 (0.5)	21.7–24.2	23.0 (0.6)
10F-South-East	24.1–26.5	25.6 (0.5)	22.4–26.2	24.7 (0.7)
10F-South-West	24.3–25.9	25.0 (0.4)	22.9–25.7	24.3 (0.5)
26F-East	23.6–25.9	24.8 (0.5)	22.4–25.7	24.0 (0.6)
26F-North-East	21.6–24.8	23.0 (0.8)	20.6–24.5	22.8 (0.7)
26F-North	22.7–24.9	24.0 (0.3)	21.4–24.2	23.2 (0.4)
42F-North-East	23.9–26.0	25.1 (0.3)	22.6–25.5	24.4 (0.4)
42F-North-West	23.2–26.1	25.2 (0.6)	21.0–25.6	24.0 (0.8)
42F-North	22.0–23.7	22.8 (0.3)	20.6–23.0	21.7 (0.5)
42F-South	22.9–25.2	24.1 (0.4)	21.3–24.5	23.0 (0.5)
B2-Workshop	22.9–27.3	25.0 (0.9)	22.3–27.3	24.5 (0.9)
Outdoor	8.8–35.7	25.5 (5.3)	-	-

**Table 3:** Linear regression equations for outdoor–indoor air temperature.

Location	C_1_	C_0_	R^2^
10F-North-East	0.22	20.16	0.78
10F-North	0.17	21.67	0.71
10F-South-East	0.16	22.50	0.72
10F-South-West	0.16	23.08	0.67
26F-East	0.21	20.32	0.76
26F-North-East	0.20	20.32	0.69
26F-North	0.16	21.45	0.69
42F-North-East	0.11	23.44	0.53
42F-North-West	0.21	20.80	0.70
42F-North	0.11	22.66	0.41
42F-South	0.11	23.29	0.46

**Table 4:** Potential air temperature set-point adjustment.

	Mean Air Temperature (24.7 °C)		Max Air Temperature (25.0 °C)	
Location	% Time < Mean	Average Set-Point Adjustment	% Time < Max	Average Set-Point Adjustment
10F-North-East	100%	0.99	100%	1.35
10F-North	82%	0.37	99%	0.64
10F-South-East	0%	NA	3%	0.18
10F-South-West	8%	0.10	58%	0.20
	Mean air temperature (23.9 °C)		Max air temperature (25.0 °C)	
26F-East	0%	NA	73%	0.42
26F-North-East	100%	0.90	100%	1.97
26F-North	48%	0.18	100%	1.00
	Mean air temperature (24.3 °C)		Max air temperature (24.7 °C)	
42F-North-East	0%	NA	4%	0.23
42F-North-West	1%	0.07	9%	0.22
42F-North	100%	1.45	100%	1.85
42F-South	82%	0.28	98%	0.62

**Table 5:** Mean range and statistics of carbon dioxide (CO_2_) levels at sampling zones.

Location	Mean Range (ppm)	Mean (std) (ppm)	% Time > 800 ppm	% Time > 1000 ppm
10F-North-East	1263–528	764 (97)	32%	2%
10F-North	1226–543	807 (86)	50%	3%
10F-South-East	1287–518	811 (95)	44%	3%
10F-South-West	1321–578	931 (80)	97%	15%
26F-East	1036–486	723 (80)	19%	0%
26F-North-East	1068–467	718 (81)	19%	1%
26F-North	975–455	683 (62)	3%	0%
42F-North-East	1009–456	696 (70)	6%	0%
42F-North-West	1085–423	601 (67)	1%	0%
42F-North	961–431	632 (65)	2%	0%
42F-South	995–460	655 (61)	2%	0%
B2-Workshop	1405–673	965 (160)	84%	40%

**Table 6:** Mean range and statistics of relative humidity at sampling zones.

Location	Mean Range (%)	Mean (std) (%)	% Time > 60%	% Time > 70%
10F-North-East	31.0–64.2	53.0 (5.1)	4%	0%
10F-North	40.6–61.4	52.0 (2.5)	1%	0%
10F-South-East	38.4–56.0	49.9 (2.5)	0%	0%
10F-South-West	32.8–54.0	47.3 (3.2)	0%	0%
26F-East	32.7–59.5	49.8 (4.8)	0%	0%
26F-North-East	32.1–74.5	56.8 (7.4)	30%	3%
26F-North	33.0–59.3	51.8 (4.5)	0%	0%
42F-North-East	34.8–52.6	47.7 (2.9)	0%	0%
42F-North-West	33.8–50.9	45.7 (2.6)	0%	0%
42F-North	32.8–61.9	53.3 (4.9)	2%	0%
42F-South	33.0–57.4	50.4 (3.6)	0%	0%

**Table 7:** Mean range and statistics of particulate matter (PM_10_ and PM_2.5_) at sampling zones.

	PM_10_		PM_2.5_	
Location	Mean Range (μg/m^3^)	Mean (std) (μg/m^3^)	Mean Range (μg/m^3^)	Mean (std) (μg/m^3^)
10F-North-East	0.0–38.3	3.7 (4.6)	0.0–41.4	4.0 (4.8)
10F-North	0.0–19.6	3.4 (3.0)	0.0–20.6	3.3 (3.0)
10F-South-East	0.0–26.5	3.6 (3.2)	0.0–29.5	3.4 (3.3)
10F-South-West	0.0–20.8	3.3 (2.7)	0.0–23.3	3.0 (2.7)
26F-East	0.0–16.8	3.3 (2.6)	0.0–16.6	2.9 (2.6)
26F-North-East	0.0–17.7	3.6 (2.7)	0.0–18.5	3.6 (2.7)
26F-North	0.0–17.1	5.6 (3.1)	0.0–17.8	5.6 (3.1)
42F-North-East	0.0–19.5	5.3 (3.9)	0.0–19.5	5.0 (3.9)
42F-North-West	0.0–13.6	3.4 (2.3)	0.0–18.4	4.4 (3.0)
42F-North	0.0–25.8	7.4 (4.9)	0.0–26.8	6.9 (4.8)
42F-South	0.0–27.6	6.4 (4.6)	0.0–28.9	6.7 (4.9)
B2-Workshop	0.3–104.0	16.4 (12.8)	0.1–105.0	16.9 (13.6)

**Table 8:** Mean range and statistics of sound pressure levels (SPL) at sampling zones.

Location	Mean Range (dBA)	Mean (std) (dBA)	% Time > 50 dBA	% Time > 60 dBA
10F-North-East	43.5–61.8	54.3 (5.4)	73%	11%
10F-North	51.5–59.5	56.2 (1.6)	100%	0%
10F-South-East	55.6–59.6	57.9 (1.1)	100%	0%
10F-South-West	59.7–63.7	62.5 (1.0)	100%	98%
26F-East	44.7–63.3	58.8 (4.5)	91%	52%
26F-North-East	30.6–60.3	44.6 (9.2)	32%	0%
26F-North	47.9–63.5	59.1 (3.1)	97%	43%
42F-North-East	31.5–59.4	54.4 (4.5)	89%	0%
42F-North-West	59.9–64.8	63.3 (1.1)	100%	99%
42F-North	52.5–69.8	62.6 (2.5)	100%	86%
42F-South	59.9–66.9	64.4 (1.6)	100%	99%
B2-Workshop	39.8–62.4	52.7 (5.32)	65%	11%

## Data Availability

Data are available upon request.
